# Extra Supplement—The Nursing Mirror

**Published:** 1890-09-20

**Authors:** 


Being the Extra Nursing Supplement of "The Hospital" Newspaper.

All Contributions for this Supplement should be addressed to the Editor, The Hospital, 140, Strand, London, W.C., and should have the word "Nursing" plainly written in left-hand top corner of the envelope.

### En Passant.

**Fresh Air.**—"From the objection which many people, especially the poor, have to fresh air, it might be a life destroyer instead of a life preserver. Never fear fresh air; it will be one of your most faithful allies in the treatment of disease. Provided a patient were kept warm enough, his chances of recovery would be much greater if he were hung outside of his ward instead of being shut up within its walls."

**Women Inspectors.**—What are the necessary qualifications for women inspectors? Miss Collette said at the discussion on workshops at the British Association, that she thought "domestic workshops" ought to be inspected by women, "but she had not yet come across any women who were fit to be inspectors." Does this mean that they are ignorant of sanitary conditions, because if so, we are of opinion that this lack of knowledge is pretty general, and exists quite as much among men as among women.

**Nursing in America.**—An English nurse writes to the editor of the *New York Nightingale* from Jersey City, N.J., and says: "I have been in this country three years, and have been private nursing for two, and have all the work that it is possible to take." . . . "The pay in America is better (than in England), notwithstanding having to pay more for board, washing, &c." Another English nurse says that she made more money in America, but that she spent much more, paying three times as much more for her room as at home.

**Bath Hospital.**—An afternoon call at the Royal United Hospital at Bath, with Mrs. Matthias, the Lady Superintendent, as guide, shows that it will be looking very smart in a short time. There is a general prevalence at present of workmen, betokening great improvements. On the top storey, a fine new ward for children is in course of completion, which will hold twenty cots, and up above that there will be a very good recreation ground for the patients. A men's surgical ward and a women's ward have been completely renovated, and, judging by the contrast between the cleaned and the uncleaned parts of the hospital, it was time alterations were made. Now the entire place is to be cleaned from top to bottom, and the walls brightened with light sage green paint, and a chocolate-coloured dado. The new polished floors contrast very favourably with the old much-scrubbed ones, which must be veritable dust collectors, but they are all going to be planed and caulked and made more healthy. "Won't it all be nice?" a nurse asked us, as we went through the ward; and we felt how "nice" it was to see money being spent in renovation, and what a difference it must make to both patient and nurse to be all day in a fresh, bright ward, instead of in one in which most of the paint had disappeared, and we wished we knew how to impress this difference on the possessors of heavy pockets. The nurses' new cubicles are good, too; each nurse has one to herself. The most dismal place in the whole building is the chapel, and it certainly is a miracle of ugliness. We came away from the hospital, feeling that enthusiasm is one of the best of virtues, and an essential to success, for the £650 which is being spent on the cleaning was the result of an A B C collection made by the wife of the President of the Hospital; and the office of fund collector is no sinecure!

**Sister Rose Gertrude.**—Report is current that Sister Rose Gertrude has resigned her charge of the Hawaiian lepers, as she does not agree with the treatment which the new manager is giving her patients.

**Menial Work.**—The *British Medical Journal*, in its issue of September 13th, dealt in a short article with the interview with Mrs. Hunter which the *Pall Mall Gazette* published on September 5th. The article combats all the charges made against the London Hospital. Anent the menial work which has to be done by the nurses, the journal states: "As to cooking done by the nurses, this amounts to little more than making tea, coffee, and cocoa, cooking an egg, or a rasher of bacon. A nurse is not very highly trained—or, perhaps, too highly trained—if she cannot render these simple services." This little comment is quite to the point; one would have thought that any service rendered on behalf of those unable to serve themselves would scarcely come under the heading of menial. "Anyone interested in nursing matters should visit the nursing home and wards of the London Hospital, and satisfy himself of the actual conditions." This applies not only to the London Hospital, but to everything. If people, instead of taking second-hand information, would go in for personal investigation, the suffering and scandal saved would reward this extra trouble.

**Native Medical Women in India.**—"By far the most important and deep-reaching of Indian social questions at this hour," says the *Daily News*, "is the rapidly progressing change in native Indian opinion on the rightful status of women." Medical science can claim no small share of this "progressing change." Medical women know that they have made it possible to relieve the misery and suffering of millions of Indian women—no slight thing to be grateful for. But though so much has been done by the example and teaching of the women of this country who, year by year, go out to India to spread medical knowledge, yet the demand is so great we cannot furnish the supply, and the great remedy lies in the medical training of native women. The last report shows that there are women in most of the medical schools. There were twenty-four women last year at the Calcutta Medical College, and at Agra seven women received diplomas and licences. In the Punjab the medical profession seems to have a great attraction. At Madras one lady took the degree of M.B. This is pleasant news to us at home, it must be untold joy to the educated women of India, who, longing for reform, saw one of their dearest hopes realized in the foundation of Lady Dufferin's Fund.

**Nursing as a Profession.**—Sometimes people account for the large number of women who enter the nursing profession year after year by describing nursing as the "latest craze," others say it is "fashionable," others again that the younger generation of nurses like the becoming attire. Of course, there may be isolated cases where one of these ridiculous reasons has been the persuasion, but can there be any foundation for the fact that anything so small and silly attracts the greater number of nurses? Is it not rather the testimony of nurses past and present which induces other women to accept service among the sick? These have discovered that there is something elevating about their work; it is not merely a source of bread-and-butter; it demands constant intelligence and care, and though it so often means self-effacement, it means satisfaction. As the nursing world gets larger and larger, and all sorts and conditions of women enter it, criticism will get keener and keener, and nurses will have to try harder than ever to show the general public that their lives are very real and earnest, and that the women who gain their livelihoods by relieving sickness and suffering, care very little for the inducements offered by becoming cap or bonnet, or mere craze or "fashion."

### Sketches of Insanity.

By FRANCIS H. WALMSLEY, M.D. (Senior Assistant Medical Officer of Leavesden Asylum).

### XI.—EPILEPTIC INSANITY (*Continued*).

EPILEPSY may co-exist with perfect sanity, yet it has, sooner or later, a fatal influence on the intellectual faculties; in proportion as the fits recur and increase in frequency, in proportion as the disease progresses, the mental powers fail, become gradually impaired, and insanity supervenes. For a certain period of their lives epileptics are industrious, complaisant, and docile; but at other times their conduct becomes suddenly modified, and presents the greatest irregularities. There is observable a singular changeableness of temper and of character, manifested by irritability, impulsiveness, and loss of self-control; the finer feelings become blunted, the most deplorable tendencies and the worse inclinations develop; those affected betray a general condition of unreasonableness, become turbulent and aggressive, pick up quarrels with those around them, exhibit lying and thieving propensities, complain of everything and of everybody, and are easily irritated from the slightest cause. Under the influence of this mental condition such patients suddenly cease their occupations or leave their homes, and wander about in an aimless, objectless fashion; they feel intensely miserable, they believe themselves to be victimised and persecuted by their relatives or their friends, they accuse all those with whom they have been in contact of being the cause of their trouble; if they have previously harboured any feelings of hatred or thoughts of revenge, these feelings are quickened and roused to a pitch of intensity which prompts them to immediate action, when they are apt, in a most sudden and unexpected manner, to commit all kinds of violence—suicide, theft, incendiarism, homicide. These attacks of epileptic furor may occur independently of the epileptic fit, and may last from a few hours to two or three days, or even a week or more; they are remarkable as a rule for the suddenness of their invasion and the suddenness of their subsidence.

No one can form an accurate notion of the sort of rage which suddenly possesses the epileptic; they appear to be seized with ideas and hallucinations of a terrifying character, they have visions almost constantly, they see frightful objects, ghosts, assassins, armed men who rush at them to kill them. When in this condition, the epileptic maniac yells and shrieks, knocks his body violently about, rushes furiously, striking whatsoever or whomsoever is in his way. During these attacks of blind, impulsive fury, the patient is scarcely, if at all, conscious of his real surroundings; when he emerges from this state of maniacal excitement, he appears to have no recollection of his painful thoughts or of his frightful hallucinations; his memory of what has occurred is exceedingly defective; sometimes he recollects fragments of what has happened as of a dream, but he can rarely call to mind all that has taken place, and perhaps forgets the main incitements entirely.

All kinds of doings after epileptic fits, from slight vagaries to homicidal actions, have one common character—*they are automatic, they are done unconsciously*.

Elaborate and highly compound actions may be performed when a patient is in this state of *mental automatism*, the cerebral activity continues its functions in an independent manner; we see individuals as if in a state of somnambulism act unconsciously, commit extravagant actions, even crimes, without having any conscious idea of the things of the external world. The following cases are illustrative of this condition:—

A carman in a state of unconscious automatism, after an epileptic seizure, drove through the most crowded parts of London without any object, but also without any accident.

A shoemaker subject to epilepsy was often furious for some time after the fits, but sensible, amiable, and industrious in the intervals. One day, when in the gloomy and morose state of mind that often precedes a fit, he met the Superintendent of the Asylum, to whom he was much attached, and stabbed him to the heart. He had not had a fit for three weeks, but the night following the homicide he had a severe fit, and for some time the attacks continued to be frequent and severe.

Again, an individual seized with epileptic mania on his wedding day killed his father-in-law with a blow from his knife, coming to himself at the end of several days. He ha not the slightest consciousness of what had taken place.

A woman subject to epileptic seizures rose up from her chair one morning with her baby in her arms and went to cut some bread for the elder child. Having got the knife in her hand for this purpose, she had an epileptic seizure, and during the unconsciousness she cut her infant's hand clean off, and was found insensible by the neighbours. She had no recollection of the circumstances after getting possession of the knife.

Many epileptics have a deep feeling of their inability to resist a superior force which holds their will in subjection, and drives them in spite of themselves to acts of violence; they say, for instance, that they are no longer themselves, that their malady drives them on, that they have within them an evil spirit which commands them to execute some deed of violence.

A peasant suffered from epilepsy from his eighth to his twenty-fifth year, when, instead of epileptic attacks, he was seized with an irresistible impulse to kill. He felt the approach of his attack sometimes for days beforehand, and begged to be restrained. "When it seizes me," he said, "I must kill someone, were it only a child." Before the attack he was very weary and depressed, could not sleep, and had slight convulsions of the limbs. Some of those who are tortured by this irresistible impulse to homicide, in order to escape their inward anxiety and dread, attempt to commit suicide.

In many individuals, the temporary intellectual disorder which succeeds an epileptic fit assumes the form of more or less marked simple maniacal excitement. The patient talks incessantly and incoherently; he moves about restlessly, and executes movements that are more disorderly than violent. An important character of epileptic mania is the absolute resemblance of all the attacks in the same patient, not only in the whole, but even in every detail; the patient expresses the same ideas, utters the same words, performs the same acts—in a word, goes through the same phenomena on the occurrence of every fresh seizure.

The termination of these maniacal attacks is, in general, as sudden as its invasion; in a few hours, sometimes even in less time, these patients return to their normal condition; they recover from their attacks like a man who wakes up after a dream or a painful nightmare. Epileptics cannot be held responsible for any act of violence perpetrated during this perfectly automatic though short-lived delirium, which they have no power to control.

*(To be continued.)*

### Appointments.

[It is requested that successful candidates will send a copy of their applications and testimonials, with date of election, to THE EDITOR, The Lodge, Porchester Square, W.]

TOYNBEE HALL.—Mrs. Perry, late of Whitechapel Infirmary, has accepted the post of matron at Toynbee Hall, Commercial-street, London, E.

WEST OF ENGLAND EYE INFIRMARY, EXETER.—Miss Georgina Kinninmont has been elected matron of this infirmary, out of 53 candidates; she was trained at Leeds General Infirmary, and University College Hospital, London, and has for the last five years been matron at Professor Jessops' Surgical Hospital at Leeds.

### Are Nurses Sweated?

The following excellent letter appeared in the *Pall Mall Gazette* of the 11th inst. We shall be glad to publish further letters on the subject.

"I have read with great interest the nursing details in your issue of September 5th. I have also worked at different periods in the London Hospital, but the conclusions I have drawn from my experiences there differ entirely from those of Miss Yatman. My case is similar to hers in that I was anxious to gain the largest and best knowledge of nursing to be had for money. I consulted my friend, Miss Beresford, matron of the City of Dublin Hospital, who advised my going to the "London," and to Miss Lückes, who, she told me, was working with administrative talent that would bring the hospital to the front as a school for nurses. My first year was 1883. I paid thirteen guineas and remained for three months. On two subsequent years I went through the same period, and have found the training I received of the greatest value to me since. I dreaded the work would be beyond my strength, and I found it very fatiguing. Work from seven to half-past nine, with only two hours off duty—no time allotted on the time-table for any meal between one o'clock p.m. and half-past nine. It sounded alarming, but I found on my arrival that the nurses sat down to a comfortable tea, but did not leave the wards. I mention these little things because in the outcry that is being made against the management of the hospital the fact seems to be overlooked that we voluntarily put ourselves into the position, taking the unpleasantness as the price to be paid in fitting ourselves to become first-rate nurses. Delicate young ladies are not material that any master hand would select for steady and continuous work. Domestic tasks that come lightly to women of a tougher fibre are a strain to them, but they work with hearty good-will; and, unreliable as their health may be, they are a valuable element in a nursing staff, and on consideration of the refinement they bring with them, and the payments that are an assistance to the hospital, Miss Lückes has hitherto welcomed their aid.

"The food I found ample and plain—abundance of meat and potatoes, good milk and bread.

"Everything has gone on since my time, and I am told that the meals are much better than they were. Complaints abounded, and I thought them bad taste when made by young ladies in the presence of a great majority of nurses who seemed content with their fare. We hear often of servants being particular and fastidious about food, but it is not an example we would wish to copy.

"Since leaving the London I have been in the Smallpox Hospital, Highgate; the British Hospital, Endell Street; in Barnwood House, Gloucester; and in Barrington's Hospital, Limerick; and I do not hesitate to say that in the London Hospital the nurses are better fed, with better cooked food, and in greater variety than in any of those I have mentioned. That hospital food to a girl with delicate digestion is sometimes trying, I know too well. The question is whether it would be desirable to spend a greater portion of the funds at the disposal of a hospital entirely supported by voluntary contributions, as is "the London," on more delicate fare.

"The night duty that Miss Yatman had to do is heavy, I admit. But let us compare it with the night duty in other hospitals. In every one of those whose name I have given there is one, and only one, nurse in charge at night of the whole building, and of every patient contained in it. I speak of them because I have worked in them. Of course inquiry would show how many more there are in the same circumstances. The funds of these small hospitals do not, as a rule, admit of a larger number of night nurses, and the work Miss Yatman must have done is not beyond the ordinary work of any woman who engages as a night nurse.

"Admitting that the hours are long and the work heavy, and that during two years life is lived in close contact with disease, in wards often overcrowded, the doctors running up two or three extra beds to meet the extreme needs of the poor population that surrounds the hospital, have we not here sufficient to account for the exceptional fact of the loss of eight nurses out of 300 in two years? Weak complaints brought against the management of the matron by individuals who have succumbed to the hard pressure of such circumstances rouse indignation, when contrasted with the whole-hearted loyalty that has carried on the noble old building for so many years.

"K. S. Hore.

"Rathwade, Bagnalstown, Co. Carlow."

### Nurses on Their Travels.

A Letter from Montreal.

Dear T.—Many thanks for newspaper cuttings, but why did you not send me the paper? I have not received a single one since I got here. I received a "Hospital" from the National Pension Fund folks two days ago; did'nt I just grab at it; everyone is so anxious to see it that there is a great demand for it; we cannot get it here, and so it came like a breath of fresh air. I shall feel grateful to the Editor for remembering me, to the end of my days. Our Superintendent says she will order it from England, but please send it to me till then. What a nice time you must have had at Marlborough House, and how pleased you must have been to see so many old friends. I hardly know Montreal yet, for I have only just returned from Ottawa, where I have been staying eight weeks. Ottawa is quite a new place, used to be an Indian village, but is now the capital. The Houses of Parliament are a splendid group of buildings, built on Parliament Hill, and surrounded by a beautiful public park; at the foot of the Hill flows the Ottawa River, which runs into the Ridian River; in the distance you can see the Chandure and Ridian Falls, a chain of hills, and some of the most lovely country. Half way down Parliament Hill and running all round it is the Lovers' Walk, a perfect ideal lovers' walk, the foliage is so thick you can hardly see through it, but here and there you catch a glimpse of sparkling water at the bottom of the hill. The odour of the numerous flowering shrubs is heavy and overpowering, the heat intense, but the singing of birds, and splashing of water all add to the charm. Everywhere is spoiled to me by the numerous creeping, crawling things that seem to swarm in every place; the natives don't seem to mind them, but I most decidedly do. Lady Stanley is building a splendid nursing institution at Ottawa for private nurses. The heat turns me greatly, makes me feel weak, and upsets my liver terribly. The winter I dread because the houses are heated, never below 70 deg., by hot water or air; all the houses are made with double windows, the outside ones made all in a piece and won't open, heavy curtains and blankets are hung over the doors, and not a single breath of fresh air let it. No wonder the people feel the cold so much and are a white-faced lot. I am sure it will make me ill; just fancy the horror of nursing a bad case of cancer without being able to have any real ventilation. I believe they don't even allow you to open the window in your own room, because it would cool the house. One of my patients was a doctor's son. I was nursing him in May and the weather beautiful; yet they made no end of fuss because I opened the window in my own room. They said, "We can always tell when an English person is in the house, we are all so cold." That is an enlightened medical man's house. What will it be in an ordinary person's? Of course now people spend most of their time cooling off on the balcony or sitting on the doorstep (the latter is quite the correct thing to do even in the best houses), and wear as little clothing as possible. The summer is trying, but the winter with the nasty, close houses I simply dread, and one cannot alter it because the people are so prejudiced they would think you were mad, and send you about your business if you attempted any kind of reform. I am much afraid that being in Rome I must do as the Romans do. Canada is a lovely place—in summer everything seems so full, rich, and clear. I am charmed with the place, but I am told it is still more lovely in winter, which seems hard to believe. I should be quite happy and content to stay here if I had some of my old friends with me, only at times a wave of longing sweeps over me to see everybody again. If only I could get an English letter at those moments, what a God send it would be!

### Everybody's Opinion.

[*Correspondence on all subjects is invited, but we cannot in any way be responsible for the opinions expressed by our correspondents. No communications can be entertained if the name and address of the correspondent is not given, or unless one side of the paper only be written on.*]

### LADY NURSES AND DOMESTIC SERVANTS.

"PRISM" writes: I have just read with unmingled pleasure the admirable article on the above subject in your last issue by "a servant." I endorse both her arguments and conclusions. Certainly there are many lady nurses in every sense of the term, but their number is steadily diminishing, and their vacancies filled by coquettes who in many cases care little for the philanthropy of their calling so long as they can don the long cloak and fall of the nurse in public. This description does not by any means apply generally, though it is painfully obvious that far too many of this class are to be found among the younger generation of nurses. The person must needs possess a prodigious fund of self-complacency and egotism who presumes that they possess educational and intellectual endowments superior to the servant class. I have had no inconsiderable experience of both classes, and am bound to say that of the two I would prefer the higher class of servants. Only last week the *Manchester Guardian* reported a case where an hospital nurse was charged with criminal libel, and the language it was proved she wrote was justly described as outrageous and abominable. For that, however, she alone was responsible, but it shows that although the nurses generally perform arduous and humane duties that must elicit the gratitude of humankind, still there are in their ranks, as there are in the ranks of every other class, many who are not only not superior to, but are absolutely inferior to many of the servant class. The first principle of the real lady nurse, as I have observed her, is humility, and if the parvenus of strings and bonnet passion copy her principle as they try to copy her station there will be no cant about superiority.

### My Staff.

Who is it I think that I shall fear,
When in the ward I first appear,
Newly attired in nurse's gear?
My Staff!

Who shows me where the things are kept,
And gives to me, with fingers deft,
The bucket, by the last "Pro." left?
My Staff!

Who bids me scrub with dexterous care
The lockers I see everywhere,
Which serve the patients as a chair?
My Staff!

Who shows me how the beds to make,
With ready hand and vigorous shake,
While I try hard to imitate?
My Staff!

Who brings me the long-handled broom,
And tells me to sweep down the room,
Because the "scrubbers" will come soon?
My Staff!

Who, when the doctors now appear,
And I am feeling strange and drear,
Gladdens me with a word of cheer?
My Staff!

Who, when I'm on an errand sent,
And understand not its intent,
Explains to me the thing that's meant?
My Staff!

Who all the day untiringly
Each task will make quite clear to me,
That otherwise so strange would be?
My Staff!

Who, when the work is done, will say,
"I'll ask the 'Sister' if you may
Go off at eight, you're tired to-day"?
My Staff!

Who, when my first exam. is o'er,
And I am a poor "Pro." no more,
Will her congratulations pour?
My Staff!

### Notes and Queries.

TO CORRESPONDENTS.—1. Questions may be written on post-cards. 2. Advertisements in disguise are inadmissible. 3. In answering a query please quote the number. 4. If a private answer is desired, a stamped addressed envelope must be forwarded.

NOTICE.—We must really ask our correspondents to be more particular in enclosing name and address. Constantly we receive queries or letters with no name attached.

### Queries.

(37) *Toxteth* will be glad to know which of the London hospitals keep their nurses for the three years, and do not send them out private nursing during that time.

### Answers.

(34) *Vanessa.*—I beg to say that children's unbleached calico night-gowns and red flannel bed jackets would be most acceptable here, viz., the Children's Hospital, Derby.—*Lady Superintendent.*

— I am sure that Miss Davis, Lady Superintendent, Children's Hospital, Shadwell, London, will be very glad to receive contributions of garments for her little patients, and I also fancy that Miss Roxburgh, Children's Infirmary, Myrtle Street, Liverpool, will be glad of some. Little flannel jackets, shawls, pinafores, socks, or stockings would all be useful.—*A. K.*

(35) Dr. William Alexander's book on Epilepsy is very good. It is published by Pentland and Co.; price 7s.—*A. K.*

### Vacancies.

THE following vacancies are announced:

### Sisters.

Seamen's Hospital Society; £30.

Royal Albert Hospital, Devonport; £25.

### Nurses.

Aberdeen Institute.

Blackheath and Richmond Institutions.

Bradford Children's Hospital; £25.

Birkdale Home, Southport.

Cheltenham Institution.

City of London Hospital for Diseases of the Chest; £20.

Dorset County Hospital; £16.

Frome Home; £22.

Hanover Institute, W.; £25.

Halifax Infirmary (assist.); £18.

Hastings Hospital (assist.).

Jessop Hospital, Sheffield; £25.

Llanelly Hospital; £25.

Levick Institution, Paris.

Leeds District Home; £25.

Mansfield Hospital, Notts; £27.

Nurses' Home, Sheffield; £25.

Norwich Home.

N.C.C. Hospital, Wisbech (assist.); £16.

Poplar and Stepney Sick Asylum, Bromley (assist.); £17 10s.

Queen Victoria Institution, Wolverhampton.

Royal Hospital, Landport, Portsmouth; £25.

Royal Nursing Association.

St. Saviour's Infirmary, S.E.; £20.

St. Helena Home.

St. John's Home, West Kirby, Cheshire; £24.

Sheffield Union; £25.

Ditto (assist.); £200.

St. George's Hospital, W.

Victoria Home, Bournemouth; £25.

West Bromwich District Hospital; £25.

Workhouse Infirmary Nursing Association.

Woolton Convalescent Institution, Liverpool (assist.).

Wirral Children's Hospital (night).

### Probationers.

Bedford General Infirmary.
Clayton Hospital, Wakefield.
General Infirmary, Hertford; £12.
Halifax Infirmary; £12.
Metropolitan Convalescent Institution; £12.
Nurses' Home, Stratford, E.
Newark Hospital.
St. Helena Home.
Sheffield Union; £10.
Wirral Children's Hospital.

### Amusements and Relaxation.

### SECOND QUARTERLY WORD COMPETITION.

Commenced July 5th, 1890, ends Sept. 27th, 1890.

Three prizes of 15s., 10s., 5s., will be given for the largest number of words derived from the words set for dissection.

Proper names, abbreviations, foreign words, words of less than four letters, and repetitions are barred; plurals, and past and present participles of verbs, are allowed. Nuttall's *Standard* dictionary only to be used.

N.B.—Word dissections must be sent in WEEKLY not later than the first post on Thursday to the *Prize Editor*, 140, Strand, W.C., arranged alphabetically, with correct total affixed.

The word for dissection for this, the TWELFTH week of the quarter, being

"SOUTHAMPTON."

| Names. | Sept. 13th. | Totals. | Names. | Sept. 13th. | Totals. |
|---|---|---|---|---|---|
| Dulcamara ......... | — | ... 503 | Jenny Wren ...... | 68 | ... 582 |
| Tinie .................. | 69 | ... 622 | Rhoda ............... | — | ... 242 |
| Henri.................. | — | ... 395 | Annabel ............ | — | ... 17 |
| Agamemnon ...... | 70 | ... 633 | Spare Moments ... | — | ... 19 |
| Patience ............ | 69 | ... 623 | Qu'appelle ......... | 65 | ... 587 |
| Brisbane ............ | — | ... 332 | Nurse.................. | — | ... 29 |
| Nurse Hilda ...... | 65 | ... 600 | A. B. C................ | — | ... 251 |
| Lightowlers ...... | 69 | ... 617 | Brickbat ............ | — | ... 77 |

### Notice to Correspondents.

N.B.—Each paper must be signed by the author with his or her real name and address. A *nom de plume* may be added if the writer does not desire to be referred to by us by his real name. In the case of all prize-winners, however, the real name and address will be published.

Competitors can enter for *all* quarterly competitions, but no competitor may take more than *one first prize* during the year.

